# Fecal Microbial Diversity in Pre-Weaned Dairy Calves as Described by Pyrosequencing of Metagenomic 16S rDNA. Associations of *Faecalibacterium* Species with Health and Growth

**DOI:** 10.1371/journal.pone.0063157

**Published:** 2013-04-30

**Authors:** Georgios Oikonomou, Andre Gustavo Vieira Teixeira, Carla Foditsch, Marcela Lucas Bicalho, Vinicius Silva Machado, Rodrigo Carvalho Bicalho

**Affiliations:** Department of Population Medicine and Diagnostic Sciences, College of Veterinary Medicine, Cornell University, Ithaca, New York, United States of America; Catalan Institute for Water Research (ICRA), Spain

## Abstract

In this study, we use barcoded pyrosequencing of the 16S rRNA gene to characterize the fecal microbiota of neonatal calves and identify possible relationships of certain microbiota profiles with health and weight gain. Fecal samples were obtained weekly from 61 calves from birth until weaning (seventh week of the calves' life). Firmicutes was the most prevalent phylum, with a prevalence ranging from 63.84% to 81.90%, followed by Bacteroidetes (8.36% to 23.93%), Proteobacteria (3.72% to 9.75%), Fusobacteria (0.76% to 5.67%), and Actinobacteria (1.02% to 2.35%). Chao1 index gradually increased from the first to the seventh postnatal week. Chao1 index was lower during the third, fourth, and fifth week of life in calves that suffered from pneumonia and were treated with antibiotics. Diarrhea incidence during the first four weeks of the calves' life was also associated with a reduction of microbial diversity during the third week of life. Increased fecal microbial diversity after the second week of life was associated with higher weight gain. Using discriminant analysis we were able to show differences in the microbiota profiles between different weeks of life, between high and low weight gain groups of calves, and between calves affected and not affected with diarrhea during the first four weeks life. The prevalence of *Faecalibacterium* spp. in the first week of life was associated with weight gain and the incidence of diarrhea, with higher prevalence being associated with higher weight gain and less diarrhea. Representative sequences from *Faecalibacterium* spp. were closely affiliated to *Faecalibacterium prausnitzii*. Results presented here provide new information regarding the intestinal microbiota of neonatal calves and its association with health and growth. Fecal microbial diversity was associated with calf age, disease status and growth rates. Results suggesting a possible beneficial effect of *Faecalibacterium* spp. on health and growth are promising.

## Introduction

The gut microbiota is known to have a role in shaping key aspects of postnatal life, such as development of the immune system [1], [2], and influencing the host's physiology, including energy balance. Transplanting the gut microbiota from normal mice into germ-free recipients increased their body fat without any increase in food consumption, suggesting that the composition of the gut microbiome can affect energy intake from the diet [3]. There is at least one type of obesity-associated gut microbiome characterized by higher relative abundance of Firmicutes or a higher Firmicutes to Bacteroidetes ratio [4], [5]. The role of the intestinal microbiota in disease has also been shown. Gut microbes serve their host by functioning as a key interface with the environment; for example, they can protect the host organism from pathogens that cause infectious diarrhea [6]. A reduced diversity of the fecal microbiota and specifically the Firmicutes in Crohn's disease patients has been reported [7]. Recently, it was demonstrated that *Faecalibacterium prausnitzii* displays anti-inflammatory action and can potentially be used in the treatment of this disease [8].

Efficient growth of pre-weaned dairy calves together with a low incidence of disease (especially diarrhea and pneumonia) is a prerequisite for optimal calf performance post-weaning and contributes to the profitability of the dairy enterprise. For every 1 kg increase of pre-weaning average daily gain, milk yield increased by 1,113 kg in the first lactation [9]. The notion that calf intestinal microbiota profiles are likely related to growth and health already exists; probiotics, bacteria with a beneficial effect on animal intestinal health, have been found to have antidiarrheal capacities and to enhance growth rates in calves [10], [11]. However, the bovine intestinal microbiota still remains largely unexplored.

Metagenomics refers to culture-independent studies of the collective set of genomes of mixed microbial communities [12]. Sequencing and analysis of hypervariable regions within the 16S rRNA gene provide a relatively rapid and cost-effective method for assessing bacterial diversity and abundance. Barcoded pyrosequencing on the Genome Sequencer FLX/454 Life Sciences platform enables a dramatic increase in throughput via parallel in-depth analysis of many samples with limited sample processing and lower costs.

The aim of this study was to use barcoded pyrosequencing to characterize the fecal microbiota of calves during the pre-weaning period (first seven weeks of life) and identify possible relationships of certain microbiota profiles with health and weight gain.

## Results

**Figure 1:**
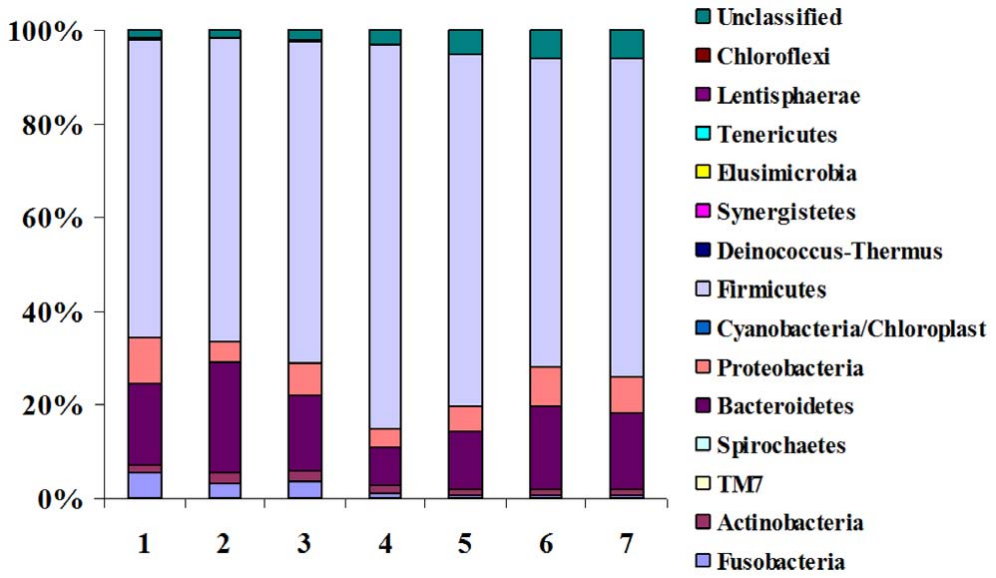
Aggregate microbiota composition at the phylum level by week of life.

Pyrosequencing produced 623,667 sequences, of which 533,208 (85.5%) were ultimately analyzed by the Ribosomal Database Project classifier (after trimming and quality control). The mean prevalence of each microbial phylum by week of calf life (from the first until the seventh week) is presented in Fig. 1. Firmicutes was the major phylum, showing a prevalence that ranged from 63.84% to 81.90%, followed by Bacteroidetes (8.36% to 23.93%), Proteobacteria (3.72% to 9.75%), Fusobacteria (0.76% to 5.67%), and Actinobacteria (1.02% to 2.35%). The Firmicutes to Bacteroidetes ratio for these seven weeks ranged from 6.15 to 46.07.

**Figure 2:**
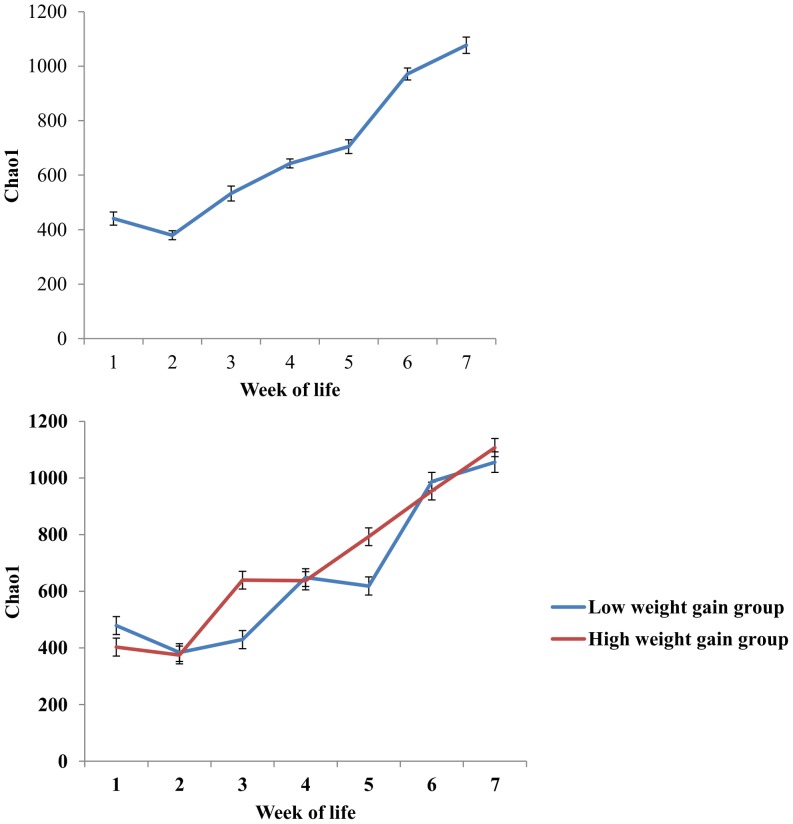
Mean (±Standard Errors) Chao1 index by week of life (top), and adjusted means (±Standard Errors) of the Chao1 index for each week of calf life and for the different weight-gain groups (low weight gain group, n = 30, high weight gain group, n = 31) (Bottom).

**Figure 3:**
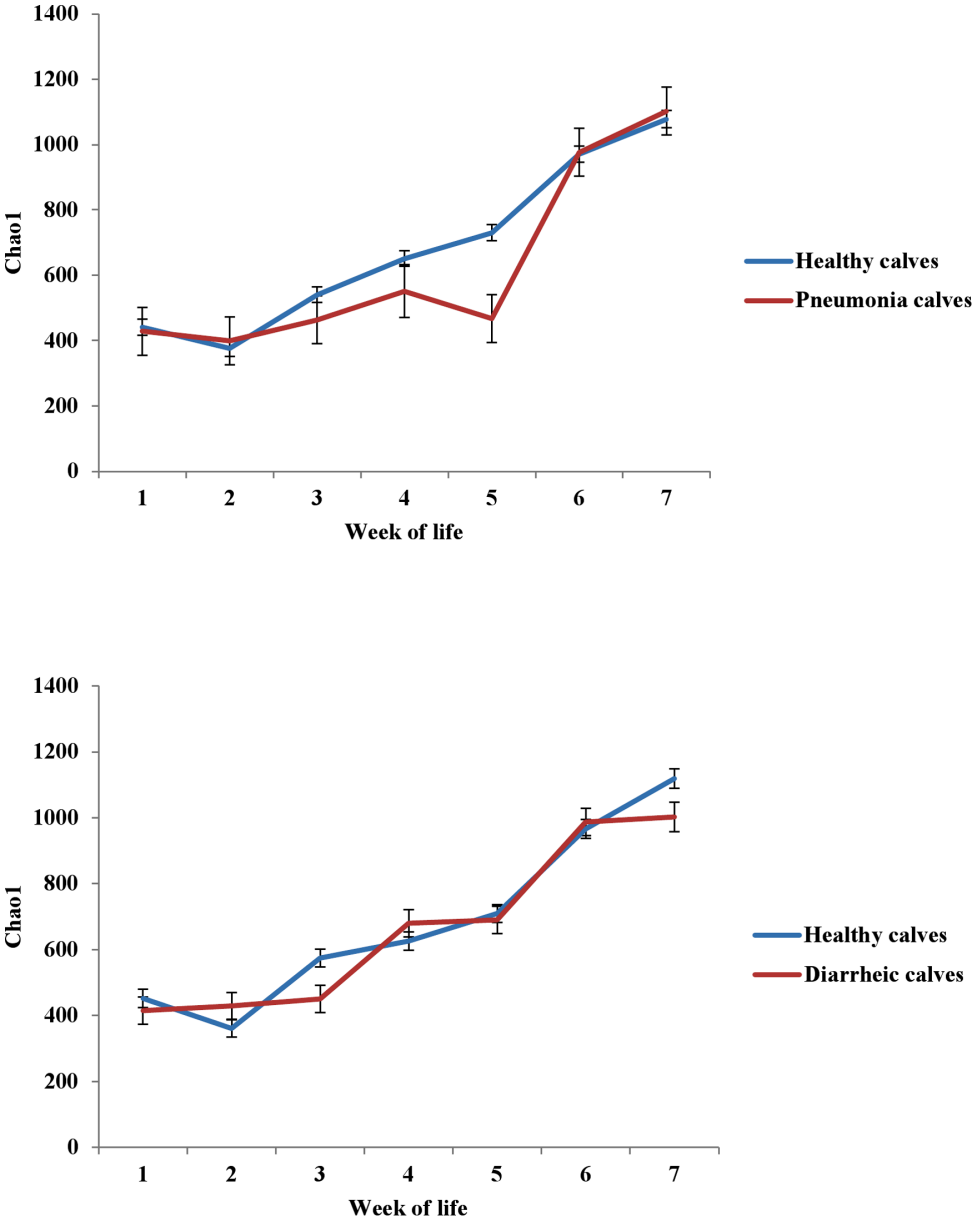
Adjusted means (±Standard Errors) of the Chao1 index for each week of calf life and for calves that were affected (n = 6) or not (n = 55) with pneumonia (top), and adjusted means (±Standard Errors) of the Chao1 index for each week of calf life for calves that were affected (n = 19) or not (n = 42) with diarrhea during the first four weeks of their life (bottom).

Mean (with standard error) Chao1 index for each week of calf life is presented in Fig. 2. Chao1 index gradually increased from the first to the seventh week of calf life. Adjusted means (with standard errors) of the Chao1 index for each week and for the different weight gain groups (low and high) are also presented in Fig. 2. The interaction between week of life and different weight gain groups was found to be statistically significant (*P<*0.05). Adjusted means (with standard errors) of the Chao1 index for each week of calf life for calves that were affected or not with pneumonia or diarrhea are presented in Fig. 3. The interactions between week of life and pneumonia or diarrhea incidence were found to be statistically significant (*P<*0.05).

**Figure 4:**
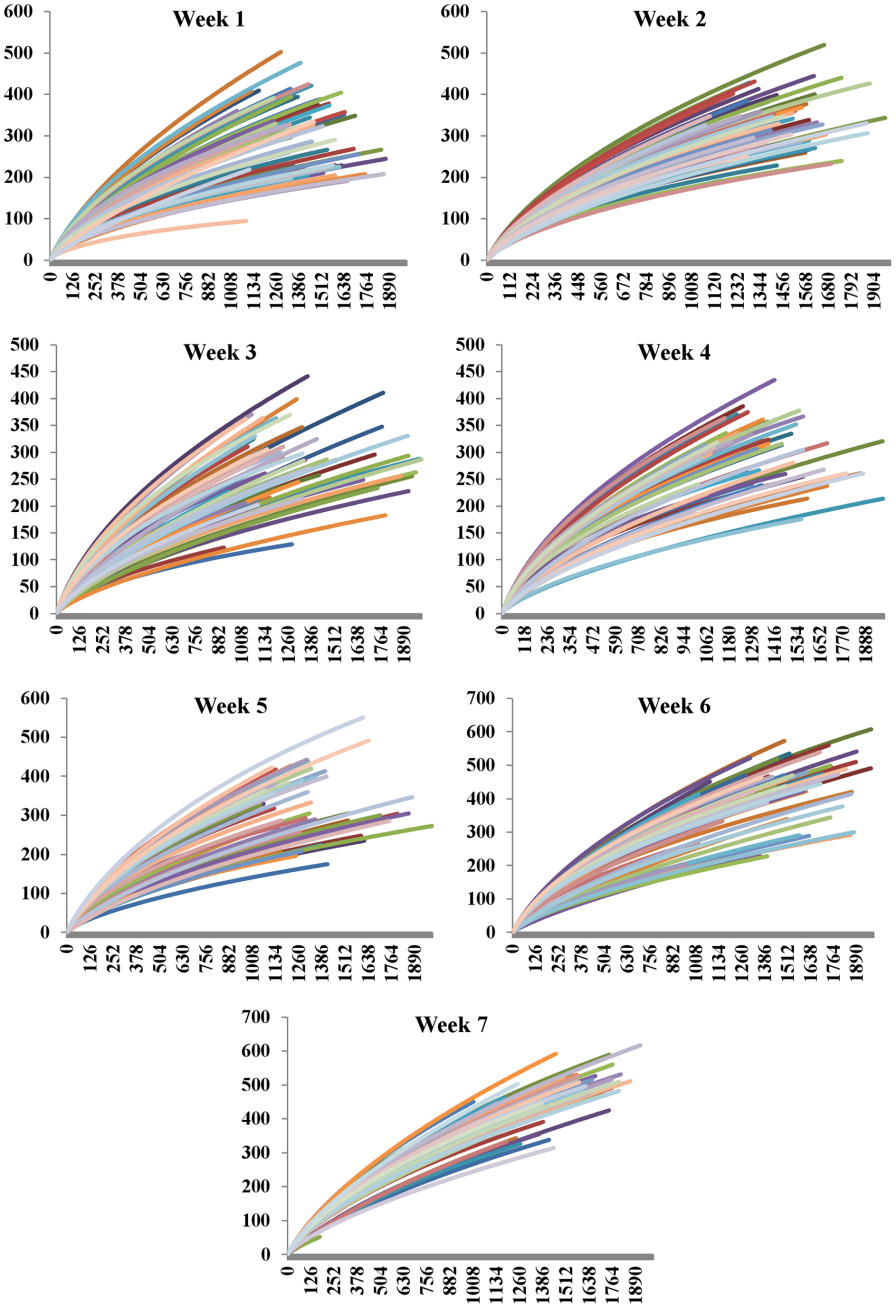
Rarefaction curves of the microbial communities of fecal samples by week of life. Operational taxonomic units (OTU) at a 0.03 distance level.

Results of a rarefaction analysis that was performed at the operational taxonomic unit (OTU) level for each different week of calf life are presented in Fig. 4. The shape of most rarefaction curves indicate that the sequencing depth reached in this study was not the maximum. This was probably a result of the large number of samples analyzed together with the great microbial diversity of the fecal samples.

**Figure 5:**
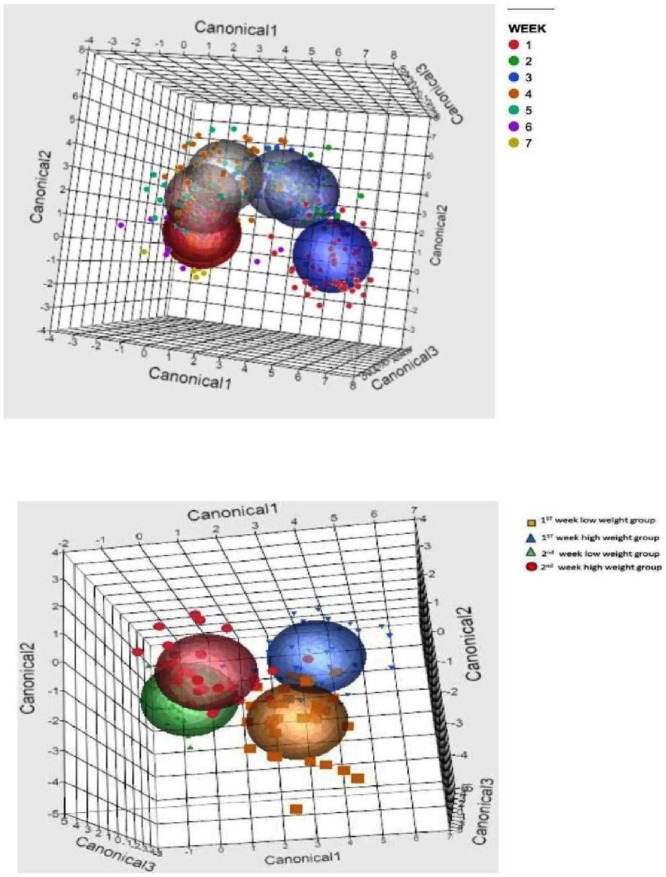
Discriminant analysis of fecal microbiomes by week of life. The microbial transition from week one (blue circle) until week seven (red circle) is illustrated by the circles, and individual data points are also depicted. Discriminant analysis was performed in JMP Pro (SAS Institute Inc., North Carolina) using bacterial genus prevalences as covariates and week of life as the categorical variable (top). Discriminant analysis of fecal microbiomes by weight gain-group and first and second week of life. First week microbiota was a strong predictor of weight gain from birth through the seventh week of life. Discriminant analysis was performed in JMP Pro (SAS Institute Inc., North Carolina) using bacterial genus prevalences as covariates and the interaction of weeks1 and 2 and weight gain (low and high) as the categorical variable (bottom).

**Figure 6:**
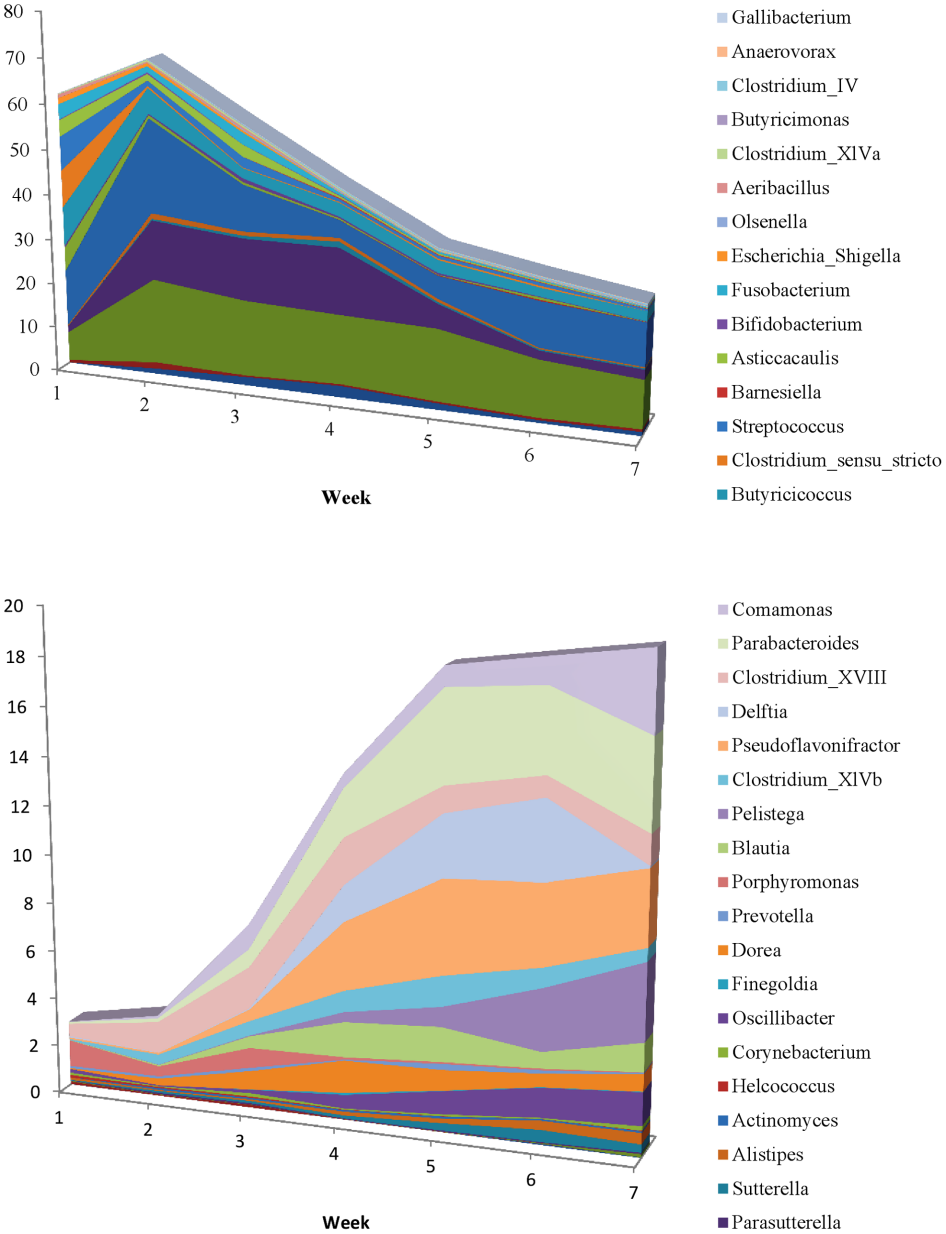
Prevalences of genera that were found to be significant for the discriminant analysis of fecal microbiome by week of life. Genera that showed higher prevalences during the first weeks of life are presented in the top graph. Genera that showed higher prevalences during the last weeks of the pre-weaning period are presented in the bottom graph.

A graphical representation of the microbial transition from week one to week seven derived from discriminant analysis that used bacterial genus prevalences as covariates and week of life as the categorical variable is depicted in Fig. 5. Prevalences of genera that were found to be significant for this analysis by week of calf life are presented in Fig. 6.

Differences in fecal microbiomes by weight gain group for the first and second week of calf life derived from discriminant analysis that used bacterial genus prevalences as covariates and the interaction of week (1 and 2) and weight gain (low and high) as the categorical variable are illustrated in Fig. 5. Certain genera prevalence during the first week of the calves' life provided significant discrimination between the high and the low weight gain groups.

**Figure 7:**
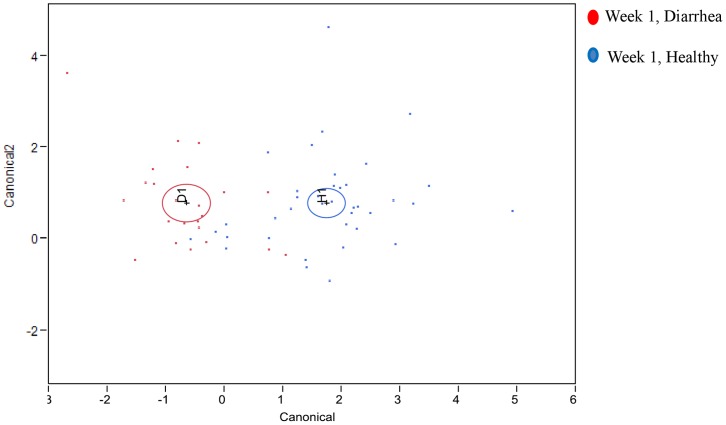
Differences in fecal microbiomes for the first week of calf life and for calves that suffered or not from diarrhea, derived from the discriminant analysis that used bacterial genus prevalences as covariates and diarrhea incidence as the categorical variable.

Differences in fecal microbiomes for the first week of calf life and for calves that were affected or not with diarrhea, derived from the discriminant analysis that used bacterial genus prevalences as covariates and diarrhea incidence as the categorical variable are illustrated in Fig. 7.

**Figure 8:**
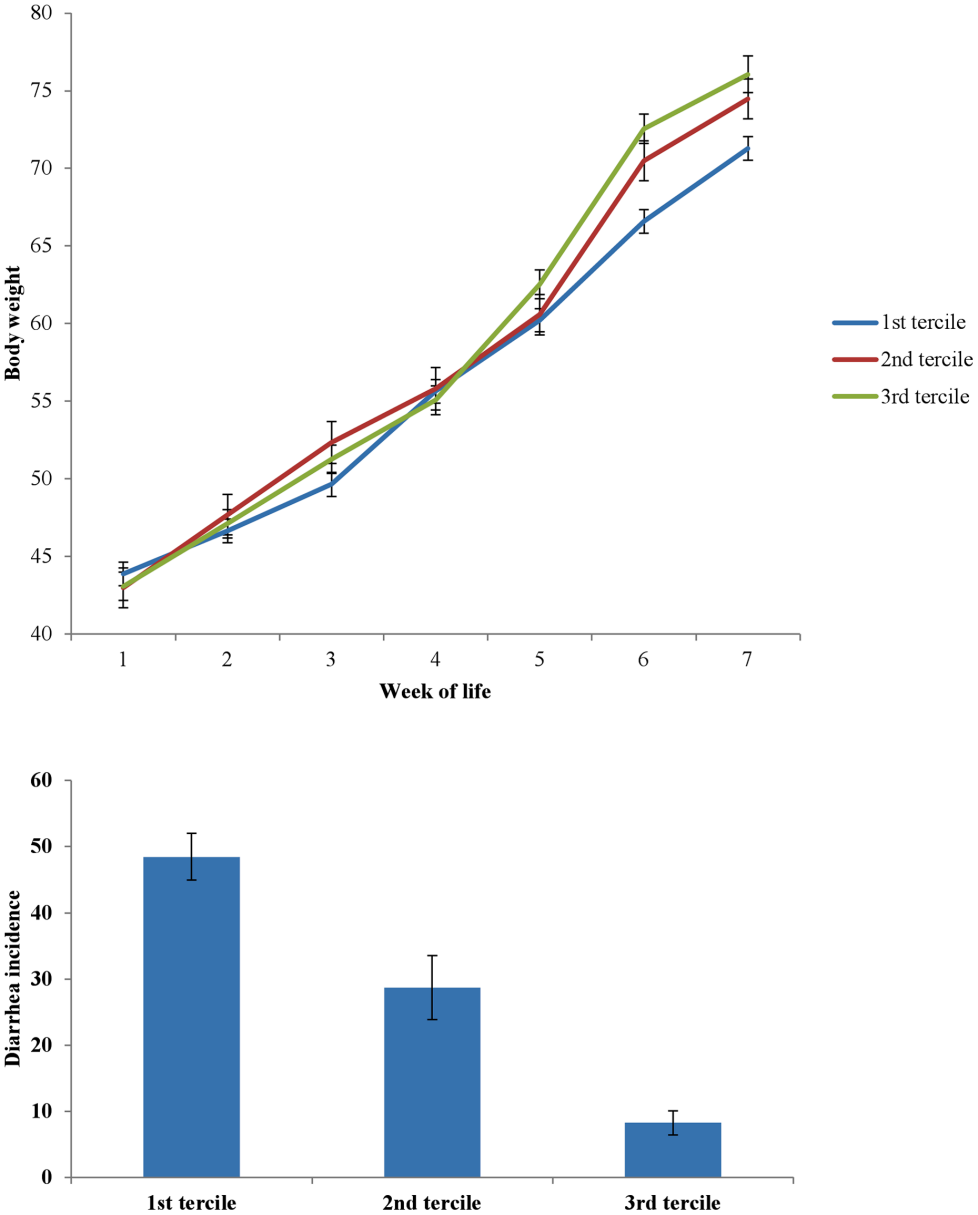
Adjusted means of body weight by week of life as well as adjusted means of diarrhea incidence for different Faecalibacterium spp. terciles. 30, 10 and 21 calves belonged to the first (lower Faecalibacterium spp. prevalence), second and third (higher Faecalibacterium spp. prevalence) tercile respectively.

Genera that were found to be significant for the discriminant analysis models, high vs. low weight-gain groups and healthy vs. diarrheic calves, were selected and further analyzed using multivariable mixed linear model (for weight gain data) or multivariable logistic regression model (for diarrhea incidence data). The statistical software MedCalc (version 12.3.0, Ostend, Belgium) was used to create terciles for each genus which were subsequently used as independent categorical variables in multivariable models. Variables were removed from the models manually in a stepwise manner and only variables with *P*-values <0.05 were kept in the final models. After the stepwise variable elimination process, the only genus that was found to be significantly associated with weight gain and diarrhea was *Faecalibacterium* spp. In detail, *Faecalibacterium* spp. prevalence during the first week of life was found to have a significant association with both body weight measurements and diarrhea incidence. Adjusted means of body weight by week of life as well as adjusted means of diarrhea incidence for different *Faecalibacterium* spp. terciles are presented in Fig. 8. Calves from the third (high prevalence) tercile had an adjusted mean body weight of 76 kg at the seventh week of life and an adjusted mean diarrhea incidence of 8.2%, while calves from the first (low prevalence) tercile had an adjusted mean body weight of 71.3 kg at the seventh week of life and an adjusted mean diarrhea incidence of 48.4%. Sequences representative of the *Faecalibacterium* spp. were found to have a 100% match with sequences from *Faecalibacterium prausnitzii.*

## Discussion

Metagenomic techniques targeting hyper-variable regions of the 16S rRNA gene are extremely powerful and generate a vast amount of data. In the present study, a total of 533,208 sequences were used in the final analysis, with an average of 1,291 sequences per sample. At present, the analysis and interpretation of this kind of data are a major challenge for researchers [13]. In the present study, we used stepwise discriminant analysis to identify bacterial genera that significantly discriminated between calves affected with diarrhea and calves not affected, as well as between calves with high weight-gain performance and those with low weight-gain performance. We were also able to identify bacterial genera that increased or decreased in prevalence as the calves aged, again by using discriminant analysis. Discriminant analysis is therefore an important tool to mine the data generated by pyrosequencing and identify the bacterial genera of interest for further analysis. Sun et al. (2010) [14] reported how discriminant analysis could be applied to derive both quantitative disease-associated microbial signatures and a detailed description of microbial community structure. Willing et al. (2010) [15] also used discriminant analysis to disclose microbial community profiles for different disease phenotypes, and our own group recently used the method to investigate microbial profiles in dairy cow mastitis [16].

We describe here changes in the intestinal microbiota of neonatal dairy calves during the first seven weeks of life. The average prevalence of Firmicutes increased from the first to the fourth week of life and then progressively decreased. A reverse pattern was observed for Bacteroidetes prevalence. It is possible that these changes reflect the gradual adaptation of the calf gastrointestinal tract first to milk consumption and later to consumption of solid feed. An observation made at the genus level supports this assumption. The prevalence of *Lactobacillus* spp. (known to be related to digestion of milk) reached a 14.74% maximum during the fourth week of calf life and then progressively decreased to reach 2.15% during the seventh week. The prevalence of *Bifidobacterium* spp. (also known to be related to digestion of milk) showed a similar pattern. It is known that the consumption of solid feed by dairy calves significantly increases after the fourth week of calf life [17].

In the present study, the microbial diversity of fecal samples steadily increased during the entire pre-weaning period, and this is in agreement with findings presented by Edrington et al. (2012) [13]. However in calves with pneumonia, the Chao1 index was lower during the third, fourth and fifth weeks of life. All pneumonia cases were diagnosed after the first week of life and then treated by parenteral administration of broad-spectrum antibiotics, which probably explains the effect of pneumonia on microbial diversity. Antibiotics usage has already been shown to affect intestinal microbiota profiles in humans [18] and swine [19]. Diarrhea incidence during the first four weeks of life was also associated with a reduction of microbial diversity during the third week of life. However, diarrhea was not treated with antibiotics and therefore this reduction of diversity was probably associated with the disease per se. It was recently reported that diversity of the intestinal microbiota is reduced in human patients with diarrhea-predominant irritable bowel syndrome [20]. On the other hand, increased fecal microbial diversity in our study was associated with higher weight gain. Interestingly, high weight-gain calves had lower Chao1 index at the first week of life and higher Chao1 index after the second week of life compared to low weight-gain calves. The fecal microbiota of high weight-gain calves during the first week of life was dominated by *Faecalibacterium* spp., a fact that likely lowered the first-week Chao1 index.

Wu et al. (2012) [21] reported that the bacterial compositions of developing versus mature rumens are strikingly different. This is also the case for the fecal microbiota even during the short pre-weaning period. Using discriminant analysis we showed here that the fecal microbiota was significantly different between samples obtained during the first week and samples obtained during the seventh week of calf life. Certain genera were reported by Wu et al. (2012) [21] to have significantly higher prevalence in the developing than the mature rumen. Some of them (*Comamonas*, *Alistipes*, *Bacteroides*, *Parabacteroides*, *Pelistega* and *Porphyromonas*) were also found in our study to have higher prevalence after the fourth week of calf life (Fig. 7).

In the present study, *Faecalibacterium* spp. prevalence during the first week of calf life was found to be significantly associated with body weight gain during the pre-weaning period. Sequences representative of *Faecalibacterium* spp. in this study were found to match with *Faecalibacterium prausnitzii* sequences. Calves from the high prevalence tercile gained 20.3% more weight until weaning compared to the low prevalence tercile calves. A recent study reported that the prevalence of *Faecalibacterium prausnitzii* in the feces of obese children was significantly higher than for non-obese children, indicating a possible energy-harvesting role of this specific microorganism in the human gut [22]. Additionally, *Faecalibacterium* spp. belong in the Firmicutes phylum, and a relatively high abundance of Firmicutes in relationship to Bacteroidetes (high Firmicutes to Bacteroidetes ratio*)* has been associated with obesity in mouse models [4]. Furthermore, the total body fat of germ-free mice inoculated with microbiota from obese mice was significantly higher than germ-free mice inoculated with microbiota from lean mice [5].

*Faecalibacterium prausnitzii* is a butyrate-producing microorganism. It has been reported that *Faecalibacterium prausnitzii* strains in human feces produced butyrate and for many strains this was associated with a net consumption of acetate [23]. Butyrate has the highest energy value per mole of the major rumen volatile fatty acids [24], is extensively metabolized by the rumen epithelium [25], and has mitogenic effects on the epithelium during development [26]. Additionally, butyrate concentrations were found to be higher in the rumen fluid of steers that showed higher feed efficiency [27].

Calves with a higher prevalence of *Faecalibacterium* spp. in the first week of life had a significantly lower incidence of diarrhea during the first four weeks. An anti-inflammatory effect of *Faecalibacterium prausnitzii*, partially due to secreted metabolites able to block NF-kB activation and IL-8 production, was recently reported, and the potential of this specific microorganism as a probiotic for Crohn's disease treatment in humans was suggested [8]. It is also known that butyrate is a key mediator in the inflammatory process in the large intestine [4], [5], while it is an important source of energy for colonic epithelial cells and may enhance the integrity of the epithelial barrier [28]. It was also recently reported that *Faecalibacterium* spp. were found decreased in dogs with acute diarrhea and active inflammatory bowel disease [29]. Admittedly, these results cannot be directly extrapolated to calves; however, they can provide a possible explanation for the association of *Faecalibacterium* spp. with diarrhea observed in our study.

Optimizing feed efficiency and disease prevention, without the use of antibiotics, are among the major goals for any farm animal production system. The results presented here provide new information regarding the intestinal microbiota of neonatal calves and its association with health and growth. Findings regarding the possible beneficial effects of *Faecalibacterium* spp. appear promising. We acknowledge that the present study is an association study and cannot show causal relationships. However, our results establish a strong foundation for future hypothesis-driven experiments to evaluate the potential of *Faecalibacterium* spp. (and most likely *Faecalibacterium prausnitzii*) as a probiotic that can enhance calf growth and health.

## Materials and Methods

### Farm and management

The study was conducted at a commercial dairy farm that milked 2,800 Holstein cows near Ithaca, NY, USA. Four L of colostrum from multiparous cows was administered to calves within 4 h of birth by esophageal tubing. Neonatal calves were transported twice daily from the maternity area to the calf barn, which was a greenhouse-type building, with 8 rows of 40 individual pens (1.7 m length by 1.2 m wide) isolated by plastic panels and bedded with a deep gravel base covered with pine shavings. Calves were kept in the same pen until weaning. Water and calf starter were available ad libitum. All calves were weaned based on age at approximately 45 days of life. Calves received daily a total of 6 L of hospital milk evenly distributed between two meals (3 L per meal). Hospital milk was pasteurized immediately before each feeding using a hospital milk high temperature short time pasteurizer (Goodnature Products). Fecal samples were collected from 61 Holstein female calves after obtaining the owner's approval. Once weekly, calf health was assessed visually by using objective criteria of appetite, fecal consistency, hydration status, respiratory effort, and attitude. Pneumonia was defined as the detection of two or more of the following clinical signs in a calf: cough, rectal temperature >39.5°C, respiratory rate >40 breaths/min, increased cranioventral lung sounds or wheezes. Calves were recorded as diarrheic when their fecal consistency was watery and fetid. 6 calves were affected with pneumonia while 19 were affected with diarrhea. All calves that were affected with pneumonia were treated with intramuscular injections of Enrofloxacin (Baytril, Bayer Animal Health) following label recommendations and all of them eventually recovered.

The body weight of the calves was measured at birth and weekly until weaning; a Waypig 15, 62-inch (157.5 cm) digital scale (Vittetoe Inc., Keota, Iowa) was used. Based on weight gain during the pre-weaning period calves were assigned to two groups. The high weight gain group (calves that gained more than 31 kg of weight, n = 31) and the low weight gain group (calves that gained less than 31 kg of weight, n = 30). Together with the weekly weight measurements, a fecal swab was obtained from each calf (a sterile cotton swab was inserted approximately 5 cm in the calves' rectum) and was frozen until used for extraction of bacterial DNA. All fecal swabs were obtained at the same time of the same date. The research protocol was reviewed and approved by the Institutional Animal Care and Use Committee of Cornell University (Protocol number: 2012-0055).

### DNA extraction

Each fecal swab was placed in 2 ml of sterile phosphate-buffered saline (PBS) and vortexed for at least two minutes. The swab was then removed and the sample centrifuged for 10 min at 13200 g. The supernatant was discarded and the remaining pellet was resuspended in 400 μl of nuclease-free water. Isolation of microbial genomic DNA was performed by using a QIAamp DNA minikit (Qiagen) according to the manufacturer's instructions. Addition of 400 μg of lysozyme and incubation for 12 h at 56°C were used to maximize bacterial DNA extraction. The DNA concentration and purity were evaluated by optical density using a NanoDrop ND-1000 spectrophotometer (NanoDrop Technologies, Rockland, DE, USA) at wavelengths of 230, 260 and 280 nm.

### PCR amplification of the V1-2 region of bacterial 16S rRNA genes

The 16S rRNA genes were individually amplified from each sample using a composite pair of primers containing a unique 10-base barcode, which was used to tag the PCR products from respective samples. The sequence of the forward primer was 5′-**CGTATCGCCTCCCTCGCGCCATCAG**NNNNNNNNNNTC *AGAGTTTGATCCTGGCTCAG*-3′: the bold sequence is the GS FLX Titanium Primer A, and the italicized sequence is the universal broadly conserved bacterial primer 27F. The sequence of the reverse primer was 5′-**CTATGCGCCTTGCCAGCCCGCTCAG**NNNNNNNNNNCA *TGCTGCCTCCCGTAGGAGT*-3′: the bold sequence is the GS FLX Titanium Primer B, and the italicized sequence is the broad-range bacterial primer 338R. The sequence NNNNNNNNNN, which is identical in the forward and reverse primer of each pair, designates the unique 10-base barcode used to tag each PCR product. A two-base linker sequence (underlined) was inserted between the barcode and the template-specific sequence to help diminish any effect the composite primer might have on the efficiency of the amplifications. PCRs were carried out in triplicate 20-μl reactions containing 0.3 μM forward and reverse primers, approximately 50****ng of template DNA and 10 μl HotStar Taq Plus Mix kit (Qiagen). A modified touchdown thermal cycling protocol was used for amplification and consisted of initial denaturation at 95°C for 2 min, followed by 30 cycles of denaturation at 95°C for 30 sec, annealing (starting at 68°C and subsequently decreased by 2°C/2 cycles until it reached 58°C, at which the 20 remaining cycles were performed) for 30 sec, extension at 72°C for 60 sec, and a final extension at 72°C for 7 min. Replicate amplicons were pooled, purified with the QIAquick PCR Purification Kit (Qiagen), and visualized by electrophoresis using 1.2% (wt/vol) agarose gels stained with 0.5 μg/ml ethidium bromide before sequencing. Blank controls, in which no DNA was added to the reaction, were performed similarly and, since these failed to produce visible PCR products, they were not analyzed further.

### Barcoded pyrosequencing of the bacterial 16S rRNA genes

Amplicons were quantified using the Quant-iT PicoGreen dsDNA Assay Kit (Invitrogen) and combined in equimolar ratios into a single tube. Pyrosequencing of the samples was carried at the Cornell University Life Sciences Core Laboratories Center using Roche 454 GS-FLX System Titanium Chemistry.

### Sequences analysis

The obtained FASTA sequences file was uploaded in the Ribosomal Database Project (RDP) pipeline initial processor that trimmed the 16S primers, tag sorted the sequences, and filtered out additional sequences of low-quality. DECIPHER was used for chimera sequences identification [30]. RDP Classifier at the RDP's Pyrosequencing Pipeline was used to assign 16S rRNA gene sequences of each sample to the new phylogenetically consistent higher-order bacterial taxonomy [31]. The produced FASTA files were also uploaded in the RDP's aligner, which aligns the sequences using the INFERNAL aligner, a Stochastic Context Free Grammar (SCFG)-based, secondary-structure aware aligner [32], and then processed by the complete linkage clustering tool (that clustered the aligned sequences into OTUs). The cluster file that was obtained from the above process was subsequently used for evaluation of sample richness and diversity through estimation of Chao1 index, again using the RDP pyrosequencing pipeline [31]. Chao1 is a nonparametric estimator of the minimum richness (number of OTUs) and is based on the number of rare OTUs (singletons and doublets) within a sample. The same cluster files were also used to obtain rarefaction curves for each sample, again using the RDP pyrosequencing pipeline.

In order to select representative sequences for *Faecalibacterium* spp. that were found to have significant effects on weight gain and diarrhea, the following procedure was used. The original FASTA file containing all the sequences was uploaded to the RDP pipeline initial processor that trimmed the 16S primers and filtered out additional sequences of low-quality. The produced file was uploaded to the RDP aligner and then processed by the complete linkage clustering tool that clustered the aligned sequences into OTUs. Finally, the dereplicate function was used to create one representative sequence for each OTU. Eventually, a new file of representative sequences was created and the RDP classifier was used again to classify them. Sequences classified as *Faecalibacterium* spp. were selected and the Basic Local Alignment Search Tool (BLASTn algorithm) from the National Center for Biotechnology Information (NCBI) (ncbi.nlm.nih.gov/BLAST/) was then used to examine the nucleotide collection (EMBL/GenBank/DDBJ/PDB) databases for sequences with high similarity to these representative sequences [33]. Sequences obtained from this project were submitted to Gen Bank (accession number: JX635481-JX643978).

### Statistical analysis

Discriminant analysis was performed in JMP Pro (SAS Institute Inc., North Carolina) using bacterial genus prevalences as covariates and week of life as the categorical variable. In this way the microbial transition from week one until week seven was illustrated. Discriminant analysis was also used to describe differences between samples' fecal microbiomes by weight-gain group during the first and second week of calf life. Bacterial genus prevalences were used as covariates and the interaction of week 1 and 2 and weight gain (low and high) as the categorical variable. Finally, discriminant analysis was used to describe differences between samples' fecal microbiomes during the first week of calf life by diarrhea incidence. Bacterial genus prevalences were used as covariates and diarrhea incidence during the pre-weaning period as the categorical variable.

**Figure 9:**
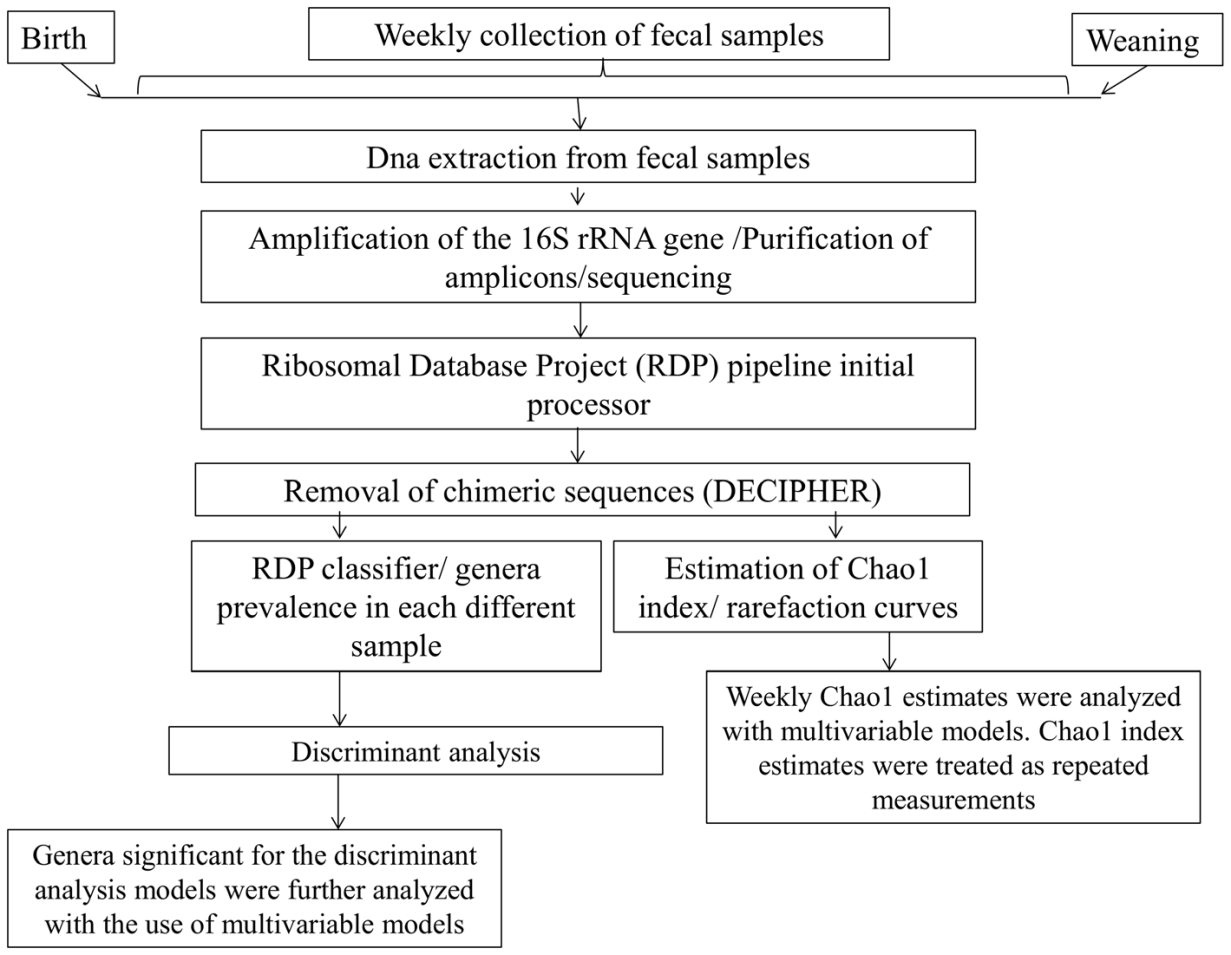
Study design.

Prevalences of genera that were found to be significant for the discriminant analysis that discriminated high and low weight-gain groups of calves or were found to be significant for the discriminant analysis that discriminated healthy and diarrheic calves were further analyzed. MedCalc (version 12.3.0, Ostend, Belgium) was used to create terciles for each genus that were subsequently used as class variables in multivariable models. Effects on weekly weight measurements were evaluated with the use of a mixed general linear model using the MIXED procedure of SAS. Body weight at birth and different genera prevalence terciles were offered to the Model. Body weight measurements were longitudinally collected and therefore treated as a repeated measurement; the error term was modeled by imposing a first-order autoregressive covariance structure to account appropriately for the within-calf correlation of weight measurements. Similar models were used to evaluate the differences in Chao1 index for calves that had or did not have pneumonia or diarrhea and for calves that belonged to the high or the low weight-gain group. Diarrhea incidence was estimated for the first four weeks of calf life. Effects on diarrhea incidence during the first four weeks of calf life were evaluated with the use of a logistic regression model that was fitted to the data using the GLIMMIX procedure of SAS. Genera prevalence terciles and body weight at birth were offered to the model. Variables were removed from the models manually in a stepwise manner and only variables with *P-*values <0.05 were kept in the final models. The design of this study and the analysis pipeline followed are illustrated in Fig. 9.
